# A nationwide trend analysis on the usage of endomyocardial biopsy

**DOI:** 10.1002/clc.24198

**Published:** 2023-12-12

**Authors:** Karsten Keller, Sebastian Göbel, Tommaso Gori, Thomas Münzel, Philip Wenzel, Lukas Hobohm

**Affiliations:** ^1^ Department of Cardiology, Cardiology I University Medical Center Mainz (Johannes Gutenberg‐University Mainz) Mainz Germany; ^2^ Center for Thrombosis and Hemostasis (CTH) University Medical Center Mainz (Johannes Gutenberg‐University Mainz) Mainz Germany; ^3^ Department of Sports Medicine, Medical Clinic VII University Hospital Heidelberg Heidelberg Germany; ^4^ German Center for Cardiovascular Research (DZHK) Partner Site Rhine Main Mainz Germany

**Keywords:** complications, endomyocardial biopsy, heart failure, mortality

## Abstract

**Background:**

Endomyocardial biopsy (EMB) is a safe procedure performed in diagnostic work‐up of cardiac disease.

**Hypothesis:**

Data regarding temporal trends of total numbers, characteristics, in‐hospital outcomes, and complications of patients undergoing EMB are sparse.

**Methods:**

The nationwide German inpatient sample (2005–2019) was used for this analysis. Patient cases of EBM during the 5‐year cycles from 2005 to 2009, 2010 to 2014, and 2015 to 2019 were compared, and temporal trends regarding total numbers and presumable major and minor EMB‐associated complications were investigated.

**Results:**

Overall, 67 745 EMB were performed in Germany 2005–2019. Total number of EMB increased from 3083 in 2005 to 5646 in 2019 (*β* 0.40 [95% confidence interval [CI] 0.37–0.43], *p* < .001). Among these EMB, 19 083 (28.2%) were performed during the period 2005–2009, 22 867 (33.7%) 2010–2014, and 25 795 (38.1%) between 2015 and 2019. The proportion of patients aged ≥70 years was highest 2015–2019 (2005–2009: 9.3%; 2010–2014: 13.8%; 2015–2019: 16.1%, *p* < .001) and the most aggravated comorbidity profile (Charlson Comorbidity Index 2.25 ± 1.93; 2.67 ± 2.14; 3.01 ± 2.29, *p* < .001) was also detected 2015–2019. Major complications occurred less often in the period 2015–2019 compared to 2005–2009 (odds ratio [OR] 0.921 [95% CI 0.893–0.950], *p* < .001), whereas minor complications were more frequently observed between 2015 and 2019 (OR 1.067 [95% CI 1.042–1.093], *p* < .001). While a decrease in major complications was detected irrespective of age, an increase in minor complications was identified only in patients between 30–59 years.

**Conclusions:**

Annual numbers of EMB increased significantly in Germany 2005–2019. Patients who underwent EMB in recent years were older and showed an aggravated comorbidity profile accompanied by fewer major complications, underscoring safety of the procedure.

## Graphical abstract

Annual numbers of endomyocardial biopsy (EMB) increased 2005–2019 (graphical illustration on the left). While patients with EMB in later years had more often severe dyspnoe and a higher Charlson comorbidity index (graphical illustration in the middle), case‐fatality remained unchanced, prevalence of minor complications increased over the years, but major complications decreased significantly (graphical illustration on the right).

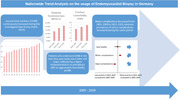

## 1 INTRODUCTION

The etiology of heart failure (HF) is in the majority of cases multifactorial,^1^ and the underlying pathologies cannot always be identified by the most frequently used diagnostic procedures such as echocardiography, coronary angiography, and cardiac magnetic resonance imaging.^2^, ^3^ Considering recent progress and improvements in development of new therapies for specific myocardial diseases, an accurate diagnosis is mandatory to choose the most appropriate and beneficial therapy for the individual patient. Endomyocardial biopsy (EMB) allows detailed tissue characterization and has been demonstrated to improve diagnostic accuracy in many cases of unexplained HF.^2^, ^4^

Historically, the first percutaneous EMB was reported by Konno and Shakakibara in the year 1963.^5^, ^6^ Subsequent modifications by Caves and Schultz^7^ and the simultaneously developed long sheath technique, improved the feasibility and safety of the EMB technique.^8^ In 2014, the radial approach was introduced with the primary aim to reduce bleeding complications.^9^ At the outset, EMB was mainly used to monitor graft rejection after cardiac transplantations.^8^, ^10^ Today, the spectrum of indications to perform EMB has considerably expanded. The established indications were recently listed and summarized in a joint consensus document by the Heart Failure Association of the European Society of Cardiology, the Heart Failure Society of America, and the Japanese Heart Failure Society.^11^ To date, several studies have reported on the safety of EMB, outlining a low rate of peri‐ and postprocedural complications, especially focusing on major complications.^12^, ^13^, ^14^, ^15^ However, data regarding EMB‐associated complications and outcomes are predominantly derived from single‐center experiences and registries.

Therefore, the objective of the present study was to investigate temporal trends regarding total numbers and complications of EMB in the German nationwide inpatient sample comprising all hospitalizations between 2005 and 2019.

## 2 MATERIAL AND METHODS

We analyzed all hospitalizations of patients undergoing EMB (OPS‐codes 1‐497.0, 1‐497.1, 1‐497.2) in Germany during the observational period between the years 2005 and 2019 (source: RDC of the Federal Statistical Office and the Statistical Offices of the federal states, diagnosis‐related groups [DRG] Statistics 2005–2019, and own calculations).

In Germany, patients' diagnoses are documented based on the coding guidelines ICD‐10‐GM (International Classification of Diseases, 10th Revision with German Modification). In addition, diagnostic, surgical, and interventional procedures are coded based on established OPS‐codes (surgery, diagnostic, and procedures codes [Operationen‐ und Prozedurenschlüssel]).^16^, ^17^, ^18^, ^19^ The Federal Statistical Office of Germany (Statistisches Bundesamt, Wiesbaden, Germany) gathers all data from hospitalized patient cases of German hospitals coded and processed according to the DRG system.^16^, ^17^, ^18^, ^19^

In the present study, we selected and included all hospitalizations of patients with performed EMB identified by the OPS‐codes 1‐497.0, 1‐497.1, 1‐497.2 during the observational period 2005–2019 in Germany. The identified and included hospitalization cases of this 15‐year observational period were subdivided according to their year of hospitalization in three 5‐year cycles: the first period includes the years 2005–2009, the second period the years 2010–2014 and the third period comprises the years 2015–2019.

We analyzed the study sample for temporal trends and differences regarding patient characteristics inclusive patients' comorbidity profile, additional treatments, and outcomes between the patients who underwent EMB during these three different 5‐year cycles.

### 2.1 Study endpoints and in‐hospital adverse events

The primary study outcomes were defined as presumable major as well as minor EMB‐associated complications. While presumable major EMB‐associated complications comprised in‐hospital death, ischemic stroke [ICD code I63], transient ischemic attack [ICD code G45], hemopericardium [ICD code I31.2], ventricular flutter/fibrillation [ICD code I49.0], implantation of a pacemaker [OPS codes 5‐377.0‐5‐377.3], implantation of cardiac resynchronization therapy [CRT; OPS code 5‐377.4], and implantation of implantable cardioverter‐defibrillator [OPS code 5‐377.5‐5‐377.7], presumable minor EMB‐associated complications included the conditions angina pectoris [ICD code I20], ventricular tachycardia [ICD code I47.2], atrioventricular block [ICD codes I44.0‐I44.3], and pericardial effusion [ICD code 31.3]). Additionally, in‐hospital death of all causes was analyzed singularly as another primary outcome. The secondary study outcome implied major adverse cardiac and cerebrovascular events (MACCE, the composite outcome of all‐cause in‐hospital death, acute myocardial infarction [ICD‐code I21], and/or ischemic stroke [ICD‐code I63]). Furthermore, the frequency of the adverse in‐hospital outcomes pneumonia (ICD‐codes J12‐J18), deep venous thrombosis and/or thrombophlebitis of the lower legs (ICD‐code I80), pulmonary embolism (ICD‐code I26), acute renal failure (ICD‐code N17), myocardial infarction (ICD‐codes I21‐I22), stroke (ischemic or hemorrhagic, ICD‐code I61‐I64), ischemic stroke [ICD‐code I63], intracerebral bleeding (ICD‐code I61), gastrointestinal bleeding (ICD‐code K92.0, K92.1, K92.2), transfusion of blood constituents (OPS‐code 8‐800), and pericardial effusion or hemopericardium (ICD‐codes I31.2 and I31.3) were investigated and assessed.

### 2.2 Definitions

Obesity was defined according to the recommendations of the World Health Organization as a body mass index ≥30 kg/m².^20^ Shock and cardiopulmonary resuscitation were both defined in accordance with current European guidelines.^21^, ^22^, ^23^

### 2.3 Ethical aspects

By German law, approval by an ethical committee and patients' informed consent were not required for the epidemiological studies of the German nationwide inpatient sample since these study did not involve direct access of the study investigators on data of individual patients, but only on aggregated summarized data.

### 2.4 Statistical methods

Temporal trends regarding annual and age‐related hospitalizations of patients with EMB and temporal trends of treatments and adverse in‐hospital outcomes of patients who underwent EMB were calculated. Linear regressions were used to assess trends overtime, and the results are shown as beta (β) with corresponding 95% confidence intervals (CI). For further temporal comparisons, we subdivided the 15‐year observational period of the present study into three different 5‐year cycles comprising the years 2005–2009, 2010–2014, and 2015–2019 and compared the three periods. Descriptive statistical comparisons of patients who underwent EMB during the three different 5‐year cycles were calculated as absolute numbers and corresponding percentages. We tested the comparison of the three different 5‐year cycles for statistical difference with the help of the Kruskal–Wallis Test.

In addition, we analyzed differences between the first and last 5‐year cycle with logistic regressions. For this purpose, we compared the last 5‐year cycle including the years 2015–2019 with the first 5‐year cycle 2005–2009 (defined as the reference). We adjusted the logistic regressions used for analyzing (I) associations of being hospitalized in a later 5‐year cycle with the different outcomes and (II) associations of being hospitalized in a later 5‐year cycle with the usage of treatments for age, sex, obesity, cancer, HF, coronary artery disease, hyperlipidaemia, chronic obstructive pulmonary disease, essential arterial hypertension, renal insufficiency (glomerular filtration rate [GFR] < 60 mL/min/1.73 m²), diabetes mellitus, and atrial fibrillation/flutter.

These comparisons between hospitalizations during the first versus last investigated 5‐year cycle computed by adjusted logistic regressions were performed for all patients with EMB regardless of age and additionally in the age subgroups <30 years, 30–59 years, and ≥60 years.

Statistical significance was presumed for *p* < .05 (two‐sided). Statistical analyses were performed with the software SPSS® (version 20.0; SPSS Inc.).

## 3 RESULTS

**Figure 1:**
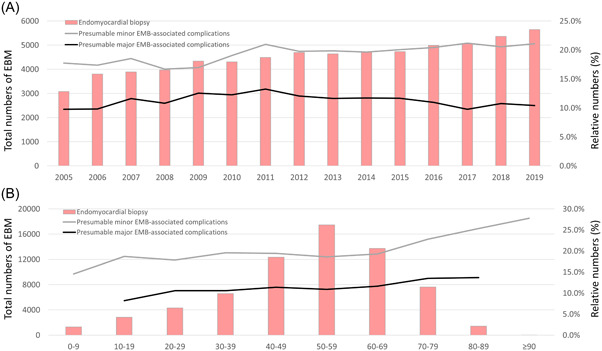
Temporal trends of performed endomyocardial biopsy (EMB) and presumable minor or major EMB‐associated complications. (A) Temporal trends of performed EMB and rate of presumable minor or major EMB‐associated complications stratified for treatment year (2005–2019). (B) Temporal trends of performed EMB and rate of presumable minor or major EMB‐associated complications stratified for age decades.

**Figure 2:**
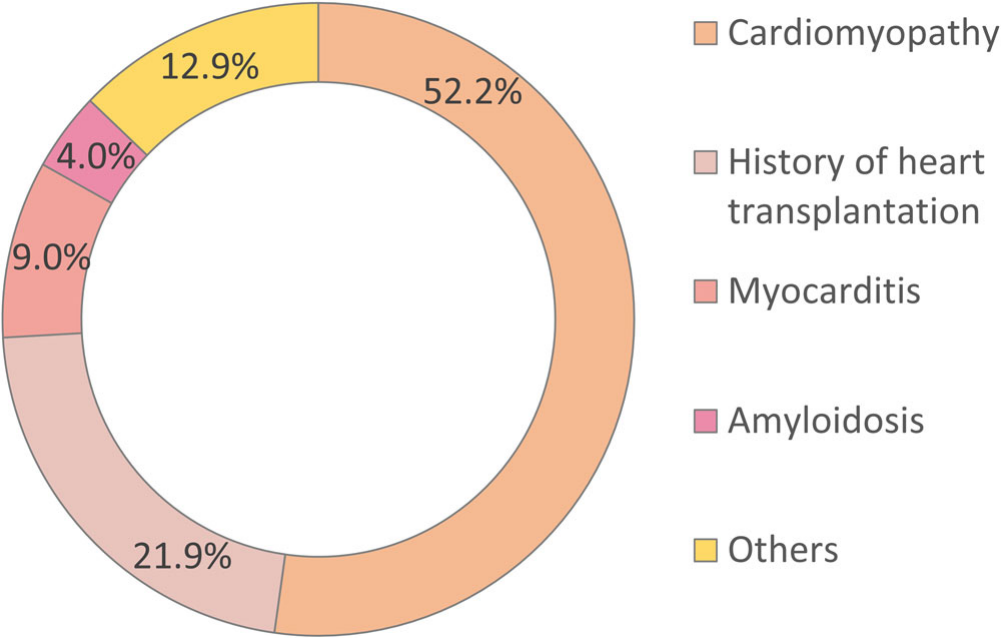
Pathologies associated with endomyocardial biopsy.

Between 2005 and 2019, 67 745 in‐hospital EMBs were documented in Germany. Annual total numbers of EMB increased from 3083 in the year 2005 to 5646 in 2019 (β 0.40 [95% CI 0.37–0.43], *p* < .001) (Figure 1A). The highest number of EMB were performed in patients between the 5th and 7th decade of life, but decreased statistically with growing age (β per age‐decade −1.44 [95% CI −1.45 to −1.42], *p* < .001) (Figure 1B). Indications to perform EMB and identified pathologies are illustrated in Figure 2. Cardiomyopathy was coded as the leading pathology in more than 50% of all EMB cases, followed by myocarditis. History of heart transplantation was the reason for performed EMB in approximately 1/5 of the cases.

### 3.1 Temporal trends of hospitalizations and patients' characteristics of patients with performed EMB

Of the aforementioned 67 745 EMB interventions, 19 083 (28.2%) were performed between 2005 and 2009, 22 867 (33.7%) between 2010 and 2014, and 25 795 (38.1%) during the period between 2015 and 2019 (Table 1).

**Table 1:** Baseline characteristics, medical history, presentation, and outcomes of the 67 745 patients with EMB stratified for the three analyzed timeframes (2005–2009, 2010–2014, and 2015–2019).

Parameters	EMB during the period 2005–2009 (19 083; 28.2%)	EMB during the period 2010–2014 (22 867; 33.7%)	EMB during the period 2015 2019 (25 795; 38.1%)	*p* Value
Age	52.0 (41.0–62.0)	53.0 (42.0–63.0)	54.0 (42.0–64.0)	**<.001**
Age ≥70 years	1770 (9.3%)	3163 (13.8%)	4156 (16.1%)	**<.001**
Female sex^a^	5323 (27.9%)	6689 (29.3%)	7494 (29.1%)	**.005**
In‐hospital stay (days)	5.0 (2.0–13.0)	6.0 (2.0–13.0)	6.0 (2.0–13.0)	**<.001**
Obesity	1633 (8.6%)	1929 (8.4%)	2068 (8.0%)	.086
NYHA functional class				
NYHA ≤ II	2481 (13.0%)	3320 (14.5%)	3796 (14.7%)	**<.001**
NYHA III	3459 (18.1%)	5481 (24.0%)	6823 (26.5%)	
NYHA IV	2527 (13.2%)	4066 (17.8%)	5468 (21.2%)	
Not classified according to NYHA classification	10 616 (55.6%)	10 000 (43.7%)	9708 (37.6%)	
Comorbidities				
Charlson Index	2.25 ± 1.93	2.67 ± 2.14	3.01 ± 2.29	**<.001**
Cancer	388 (2.0%)	497 (2.2%)	555 (2.2%)	.572
Coronary artery disease	4946 (25.9%)	6954 (30.4%)	8081 (31.3%)	**<.001**
Cardiomyopathy	9134 (47.9%)	12 439 (54.4%)	13 806 (53.5%)	**<.001**
Heart failure	9193 (48.2%)	13 364 (58.4%)	16 661 (64.6%)	**<.001**
Myocarditis	1689 (8.9%)	1909 (8.3%)	2527 (9.8%)	**<.001**
Atrial fibrillation/flutter	3312 (17.4%)	4632 (20.3%)	6067 (23.5%)	**<.001**
Peripheral artery disease	254 (1.3%)	362 (1.6%)	450 (1.7%)	**.002**
Amyloidosis	419 (2.2%)	735 (3.2%)	1561 (6.1%)	**<.001**
Chronic obstructive pulmonary disease	938 (4.9%)	1125 (4.9%)	1298 (5.0%)	.802
Essential arterial hypertension	6284 (32.9%)	8510 (37.2%)	10 156 (39.4%)	**<.001**
Hyperlipidemia	4677 (24.5%)	5918 (25.9%)	6923 (26.8%)	**<.001**
Diabetes mellitus	2752 (14.4%)	3668 (16.0%)	4099 (15.9%)	**<.001**
Renal impairment (acute and chronic kidney impairment)	5257 (27.5%)	5752 (25.2%)	7494 (29.1%)	**<.001**
Chronic renal insufficiency (glomerular filtration rate <60 mL/min/1.73 m²)	1811 (19.5%)	3499 (15.3%)	4697 (18.2%)	**<.001**
History of heart transplantation	5438 (28.5%)	4814 (21.1%)	4564 (17.7%)	**<.001**
Adverse events during hospitalization				
Presumable EMB‐associated complications				
Presumable major EMB‐associated complications	2101 (11.0%)	2788 (12.2%)	2760 (10.7%)	**<.001**
In‐hospital death	269 (1.4%)	334 (1.5%)	451 (1.7%)	**.006**
Ischemic stroke	130 (0.7%)	230 (1.0%)	386 (1.5%)	**<.001**
Transient ischemic attack	47 (0.2%)	55 (0.2%)	58 (0.2%)	.886
Hemopericardium	123 (0.6%)	153 (0.7%)	246 (1.0%)	**<.001**
Ventricular flutter/fibrillation	262 (1.4%)	398 (1.7%)	521 (2.0%)	**<.001**
Implantation of pacemaker	289 (1.5%)	325 (1.4%)	280 (1.1%)	**<.001**
Implantation of CRT	16 (0.08%)	26 (0.11%)	38 (0.15%)	.149
Implantation of implantable cardioverter‐defibrillator	1250 (6.6%)	1649 (7.2%)	1214 (4.7%)	**<.001**
Presumable minor EMB‐associated complications	3327 (17.4%)	4542 (19.9%)	5333 (20.7%)	**<.001**
Angina pectoris	934 (4.9%)	883 (3.9%)	821 (3.2%)	**<.001**
Ventricular tachycardia	1313 (6.9%)	1792 (7.8%)	2168 (8.4%)	**<.001**
Atrioventricular block	652 (3.4%)	924 (4.0%)	1158 (4.5%)	**<.001**
Pericardial effusion	757 (4.0%)	1408 (6.2%)	1790 (6.9%)	**<.001**
Other adverse in‐hospital events				
MACCE	649 (3.4%)	1046 (4.6%)	1337 (5.2%)	**<.001**
Stroke (ischemic or hemorrhagic)	163 (0.9%)	276 (1.2%)	440 (1.7%)	**<.001**
Myocardial infarction	289 (1.5%)	562 (2.5%)	595 (2.3%)	**<.001**
Cardiopulmonary resuscitation	377 (2.0%)	478 (2.1%)	566 (2.2%)	.278
Pericardial effusion or hemopericardium	869 (4.6%)	1551 (6.8%)	2016 (7.8%)	**<.001**
Pulmonary embolism	103 (0.5%)	132 (0.6%)	225 (0.9%)	**<.001**
Deep venous thrombosis and/or thrombophlebitis	121 (0.6%)	210 (0.9%)	300 (1.2%)	**<.001**
Pneumonia	742 (3.9%)	1236 (5.4%)	1811 (7.0%)	**<.001**
Acute renal failure	586 (3.1%)	1015 (4.4%)	2154 (8.4%)	**<.001**
Shock	382 (2.0%)	729 (3.2%)	1254 (4.9%)	**<.001**
Intracerebral bleeding	16 (0.08%)	34 (0.15%)	57 (0.22%)	**.001**
Gastrointestinal bleeding	61 (0.32%)	96 (0.42%)	146 (0.57%)	**<.001**
Transfusion of blood constituents	1655 (8.7%)	1972 (8.6%)	2136 (8.3%)	.250
Treatment				
PCI	404 (2.1%)	623 (2.7%)	862 (3.3%)	**<.001**
PCI with bare metal stent	184 (1.0%)	171 (0.7%)	25 (0.1%)	**<.001**
PCI with drug‐eluting stent	143 (0.7%)	321 (1.4%)	678 (2.6%)	**<.001**
Coronary artery bypass graft	36 (0.2%)	50 (0.2%)	23 (0.1%)	**.001**
TAVR	0 (0%)	9 (0.04%)	36 (0.14%)	**<.001**
Percutaneous edge‐to‐edge mitral regurgitation valve repairs with the MitraClip® implantation	0 (0%)	11 (0.05%)	42 (0.16%)	**<.001**
Heart valve surgery	81 (0.4%)	116 (0.5%)	85 (0.3%)	**.010**
Implantation of pacemaker	289 (1.5%)	325 (1.4%)	280 (1.1%)	**<.001**
Implantation of CRT	16 (0.08%)	26 (0.11%)	38 (0.15%)	.149
Implantation of implantable cardioverter‐defibrillator	1250 (6.6%)	1649 (7.2%)	1214 (4.7%)	**<.001**
Catheter ablation for the treatment of cardiac arrhythmias	197 (1.0%)	334 (1.5%)	588 (2.3%)	**<.001**
Ventricular assist device	260 (1.4%)	455 (2.0%)	505 (2.0%)	**<.001**
Heart transplantation	948 (5.0%)	919 (4.0%)	895 (3.5%)	**<.001**
Pericardial puncture	199 (1.0%)	329 (1.4%)	420 (1.6%)	**<.001**

*Note*: Bold values indicate statistical significance at *p* < .05 (two‐sided).

Abbreviations: CRT, cardiac resynchronization therapy; EMB, endomyocardial biopsy; MACCE, major adverse cardio‐cerebral‐vascular events; NYHA, New York Heart Association; PCI, percutaneous coronary intervention; TAVR, transcatheter aortic valve replacement.

^a^ Information available for 67 742 patients.

Although the median age of patients was only slightly higher in the later 5‐year cycles, the proportion of patients aged ≥70 years was approximately 7% higher in the last observational period (2015–2019) compared with the first observational period (2005–2009) (Table 1). Patients undergoing EMB in later years (2015–2019) were more symptomatic as indicated by a higher NYHA‐functional class. Importantly, patients who underwent an EMB in later 5‐year cycles had an unfavorable comorbidity profile, reflected by a higher Charlson Comorbidity Index and an increasing prevalence of cardiovascular comorbidities (Table 1). In contrast, history of heart transplantation as the primary cause of EMB decreased from 28.5% of all EMB during the timeframe 2005–2009 to 17.7% during the period 2015–2019 (Table 1).

### 3.2 Temporal trends of complications, in‐hospital case‐fatality, and other adverse outcomes and interventional/surgical treatments of patients with performed EMB

The prevalence of major complications was highest in the second 5‐year cycle (12.4%, 2010–2014) und lowest in the last 5‐year cycle (2015–2019) with 10.7% (Table 1). In contrast, the prevalence of minor complications increased constantly from 17.4% in the first to 20.7% in the last investigated 5‐year cycle. In‐hospital case‐fatality demonstrated a slightly higher value in 2015–2019 (1.7%), compared to the first (1.4%) and second (1.5%) 5‐year cycles (*p* = .006). The frequency of ischemic stroke increased also overtime (0.7%, 1.0%, 1.5%, *p* < .001) (Table 1).

To analyze annual temporal trends, we calculated unadjusted linear regressions. While total annual numbers of major complications did not changed significantly overtime during the observational period (β −0.0004 [95% CI −0.0010 to −0.0001], *p* = .142), annual numbers of presumable minor complications (β 0.0028 [95% CI 0.0021–0.0035], *p* < .001), and annual numbers of in‐hospital case‐fatality (β 0.0004 [95% CI 0.0002–0.0006], *p* = .001) increased slightly from 2005 to 2019 (Figure 1A).

The total numbers of major complications (β 0.0056 [95% CI 0.0043–0.0070], *p* < .001) and minor complications (β 0.0066 [95% CI 0.0049–0.0083], *p* < .001) as well as the annual numbers of in‐hospital case‐fatality (β 0.0018 [95% CI 0.0013–0.0024], *p* < .001) increased with growing age‐decade (Figure 1B).

In addition, MACCE rate increased overtime in light of aggravated patient characteristics (Table 1). Ventricular assist devices were distinctly more often implanted in the patients who underwent EMB during the hospitalizations in the two later 5‐year cycles than during 2005–2009 (2005–2009: 260; 2010–2014: 455; 2015–2019: 505). In contrast, heart transplantation surgeries were less often performed during later years (2005–2009: 948; 2010–2014: 919; 2015–2019: 895) (Table 1).

### 3.3 Comparison of the last versus first 5‐year cycle (2015–2019 vs. 2005–2009) regarding complications, adverse in‐hospital outcomes independently of age, sex, and comorbidities

For these analyses, we compared the last 5‐year cycle (2015–2019) versus the first 5‐year cycle 2005–2009 (defined as the reference) and adjusted the logistic regressions for age, sex, and comorbidities.

EMB performed during the years 2015–2019 was independently associated with lower prevalence of major complications (OR 0.921 [95% CI 0.893–0.950], *p* < .001), but higher rate of minor complications (OR 1.067 [95% CI 1.042–1.093], *p* < .001) and unchanged in‐hospital case‐fatality (OR 0.993 [95% CI 0.918–1.074], *p* = .856) (Table 2).

**Table 2:** Comparison of first and last investigated 5‐year cycles (years 2005–2009 vs. 2015–2019) of patients with myocardial biopsy (univariate and multivariate logistic regression model).

	Univariate regression model		Multivariate regression model^a^	
	OR (95% CI)	*p* Value	OR (95% CI)	*p* Value
Presumable major EMB‐associated complications	0.984 (0.955–1.014)	.296	0.921 (0.893–0.950)	**<.001**
Presumable minor EMB‐associated complications	1.106 (1.081–1.133)	**<.001**	1.067 (1.042–1.093)	**<.001**
In‐hospital death	1.116 (1.034–1.204)	**.005**	0.993 (0.918–1.074)	.856
MACCE	1.246 (1.188–1.307)	**<.001**	1.136 (1.081–1.193)	**<.001**
Myocardial infarction	1.239 (1.154–1.330)	**<.001**	1.148 (1.067–1.235)	**<.001**
Pneumonia	1.366 (1.308–1.427)	**<.001**	1.248 (1.193–1.305)	**<.001**
Deep venous thrombosis or thrombophlebitis	1.358 (1.221–1.510)	**<.001**	1.268 (1.138–1.412)	**<.001**
Pulmonary embolism	1.273 (1.133–1.431)	**<.001**	1.206 (1.071–1.358)	**.002**
Acute renal failure	1.696 (1.619–1.777)	**<.001**	1.493 (1.423–1.567)	**<.001**
Shock	1.582 (1.492–1.676)	**<.001**	1.455 (1.371–1.544)	**<.001**
Cardiopulmonary resuscitation	1.055 (0.988–1.127)	.111	0.961 (0.899–1.029)	.255
Stroke (ischemic or hemorrhagic)	1.419 (1.297–1.553)	**<.001**	1.300 (1.186–1.426)	**<.001**
Ischemic stroke	1.488 (1.347–1.644)	**<.001**	1.363 (1.232‐1.509)	**<.001**
Transient ischemic attack	0.955 (0.788–1.156)	.635	0.914 (0.752–1.110)	.365
Intracerebral bleeding	1.625 (1.231–2.144)	**<.001**	1.494 (1.128–1.978)	**.005**
Gastrointestinal bleeding	1.332 (1.147–1.547)	**<.001**	1.168 (1.002–1.362)	**.047**
Transfusion of blood constituents	0.975 (0.943–1.008)	.140	0.853 (0.823–0.884)	**<.001**
Pericardial effusion or hemopericardium	1.333 (1.280–1.389)	**<.001**	1.245 (1.194–1.298)	**<.001**
Hemopericardium	1.241 (1.112–1.384)	**<.001**	1.086 (0.971–1.215)	.150
Pericardial effusion	1.312 (1.259–1.367)	**<.001**	1.250 (1.199–1.304)	**<.001**
Ventricular flutter/fibrillation	1.211 (1.126–1.302)	**<.001**	1.211 (1.125–1.303)	**<.001**
Angina pectoris	0.799 (0.761‐0.838)	**<.001**	0.777 (0.740–0.817)	.777
Ventricular tachycardia	1.111 (1.073–1.151)	**<.001**	1.073 (1.035–1.112)	**<.001**
Atrioventricular block	1.149 (1.095–1.206)	**<.001**	1.113 (1.060–1.169)	**<.001**
Treatment				
PCI	1.264 (1.191–1.342)	**<.001**	1.148 (1.077–1.223)	**<.001**
Coronary artery bypass graft	0.687 (0.529–0.893)	**<.001**	0.597 (0.455–0.782)	**<.001**
TAVR	‐	**‐**	‐	**‐**
Percutaneous edge‐to‐edge mitral regurgitation valve repairs with the MitraClip® implantation	‐	**‐**	‐	**‐**
Heart valve surgery	0.881 (0.756–1.026)	.102	0.806 (0.689–0.943)	**.007**
Implantation of pacemaker	0.845 (0.778–0.918)	**<.001**	0.824 (0.757–0.898)	**<.001**
Implantation of CRT	1.326 (0.990–1.776)	.058	1.155 (0.857–1.559)	.344
Implantation of implantable cardioverter‐defibrillator	0.839 (0.806–0.874)	**<.001**	0.798 (0.765–0.832)	**<.001**
Catheter ablation for the treatment of cardiac arrhythmias	1.495 (1.379–1.622)	**<.001**	1.508 (1.388–1.638)	**<.001**
Ventricular assist device	1.202 (1.115–1.297)	**<.001**	1.075 (0.994–1.161)	.069
Heart transplantation	0.829 (0.791–0.869)	**<.001**	0.690 (0.656–0.725)	**<.001**
Pericardial puncture	1.253 (1.151–1.364)	**<.001**	1.193 (1.093–1.301)	**<.001**

*Note*: The first 5‐year cycle (2005–2009) was determined as the reference cycle. Bold values indicate statistical significance at *p* < .05 (two‐sided).

Abbreviations: CI, confidence interval; CRT, cardiac resynchronization therapy; EMB, endomyocardial biopsy; MACCE, major adverse cardiac and cerebrovascular events; OR, odds ratio; PCI, percutaneous coronary intervention; TAVR, transcatheter aortic valve replacement.

^a^ Adjusted for age, sex, obesity, cancer, heart failure, coronary artery disease, hyperlipidemia, chronic obstructive pulmonary disease, essential arterial hypertension, renal insufficiency (GFR < 60 mL/min/1.73 m²), diabetes mellitus, and atrial fibrillation/flutter.

In addition, EMB procedures performed during the last 5‐year cycle (2015–2019) were independently associated with higher MACCE rate (OR 1.136 [95% CI 1.081–1.193], *p* < .001), mainly driven by increased numbers of ischemic stroke (OR 1.300 [95% CI 1.186–1.426], *p* < .001) (Table 2). Further time‐trends of interventional/surgical treatments showed the following changes: While total numbers of ventricular assist devices remained in their frequency statistically unchanged (*p* = .069) after adjustment for age, sex, and comorbidities, heart transplantations decreased independently (OR 0.690 [95% CI 0.656–0.725], *p* < .001) from the first to the last investigated 5‐year cycle (Table 2).

In the age‐dependent analyses, occurrence of major complication decreased independently 2015–2019 versus 2005–2009 in the age groups <30 years (OR 0.837 [95% CI 0.763–0.918], *p* < .001), 30–59 years (OR 0.926 [95% CI 0.887–0.966], *p* < .001) and ≥60 years (OR 0.947 [95% CI 0.897–0.999], *p* = .047), while minor complication increased in patients aged 30–59 years (OR 1.105 [95% CI 1.069–1.144], *p* < .001), but not in the age groups ≥60 years (OR 1.028 (0.985–1.073), *p* = .205) and <30 years (OR 1.001 [95% CI 0.931–1.075], *p* = .987) (Table 3).

**Table 3:** Comparison of first and last investigated 5‐year cycles (years 2005–2009 vs. 2015–2019) of patients with myocardial biopsy (univariate and multivariate logistic regression model) stratified for age groups <30 years, 30–59 years, and ≥60 years.

	Age group <30 years				Age group 30–59 years				Age group ≥60 years			
	Univariate regression model		Multivariate regression model^a^		Univariate regression model		Multivariate regression model^a^		Univariate regression model		Multivariate regression model^a^	
	OR (95% CI)	*p* Value	OR (95% CI)	*p* Value	OR (95% CI)	*p* Value	OR (95% CI)	*p* Value	OR (95% CI)	*p* Value	OR (95% CI)	*p* Value
Presumable major EMB‐associated complications	0.861 (0.787–0.943)	**.001**	0.837 (0.763–0.918)	**<.001**	0.976 (0.937–1.017)	.252	0.926 (0.887–0.966)	**<.001**	1.031 (0.980–1.084)	.245	0.947 (0.897–0.999)	**.047**
Presumable minor EMB‐associated complications	1.012 (0.943–1.086)	**.734**	1.001 (0.931–1.075)	.987	1.135 (1.098–1.173)	**<.001**	1.105 (1.069–1.144)	**<.001**	1.100 (1.056–1.146)	**<.001**	1.028 (0.985–1.073)	.205
In‐hospital death	0.990 (0.795–1.232)	.926	0.969 (0.774–1.212)	.780	1.070 (0.955–1.199)	.244	0.977 (0.869–1.099)	.699	1.171 (1.042–1.317)	**.008**	1.012 (0.893–1.145)	.856
MACCE	1.061 (0.914–1.231)	.437	1.022 (0.877–1.191)	.779	1.272 (1.188–1.363)	**<.001**	1.195 (1.114–1.282)	**<.001**	1.239 (1.150–1.336)	**<.001**	1.105 (1.021–1.196)	**.013**
Myocardial infarction	1.212 (0.925–1.589)	.164	1.252 (0.946–1.656)	.116	1.314 (1.184–1.459)	**<.001**	1.280 (1.150–1.425)	**<.001**	1.142 (1.029–1.266)	**.012**	1.008 (0.904–1.124)	.883
Pneumonia	1.189 (1.058–1.338)	**.004**	1.167 (1.033–1.319)	**.013**	1.423 (1.340–1.511)	**<.001**	1.307 (1.229–1.390)	**<.001**	1.354 (1.254–1.463)	**<.001**	1.222 (1.127–1.325)	**<.001**
Deep venous thrombosis or thrombophlebitis	1.312 (0.958–1.799)	.091	1.289 (0.935–1.777)	.121	1.546 (1.338–1.787)	**<.001**	1.434 (1.238–1.661)	**<.001**	1.105 (0.921–1.324)	.283	1.045 (0.865–1.263)	.649
Pulmonary embolism	1.662 (1.058‐2.613)	**.028**	1.605 (1.015–2.536)	**.043**	1.349 (1.157–1.573)	**<.001**	1.268 (1.085–1.483)	**.003**	1.091 (0.894–1.329)	.391	1.059 (0.861–1.302)	.588
Acute renal failure	1.310 (1.141–1.504)	**<.001**	1.236 (1.071–1.428)	**.004**	1.717 (1.611–1.830)	**<.001**	1.557 (1.457–1.663)	**<.001**	1.778 (1.642–1.926)	**<.001**	1.549 (1.425–1.683)	**<.001**
Shock	1.593 (1.370–1.852)	**<.001**	1.570 (1.344–1.834)	**<.001**	1.571 (1.456–1.696)	**<.001**	1.446 (1.336–1.564)	**<.001**	1.628 (1.455–1.820)	**<.001**	1.495 (1.332–1.678)	**<.001**
Cardiopulmonary resuscitation	0.805 (0.688–0.941)	**.006**	0.784 (0.668–0.921)	**.003**	1.101 (1.003–1.209)	**.043**	1.011 (0.918–1.113)	.826	1.147 (1.018–1.292)	**.024**	1.004 (0.886–1.137)	.950
Stroke (ischemic or hemorrhagic)	1.151 (0.899–1.474)	.263	1.099 (0.852–1.416)	.467	1.449 (1.288–1.631)	**<.001**	1.319 (1.170–1.487)	**<.001**	1.517 (1.274–1.805)	**<.001**	1.453 (1.213–1.739)	**<.001**
Ischemic stroke	1.120 (0.853–1.469)	.415	1.046 (0.791–1.384)	.751	1.523 (1.336–1.735)	**<.001**	1.388 (1.216–1.585)	**<.001**	**1**.632 (1.344–1.981)	**<.001**	1.550 (1.269–1.894)	**<.001**
Transient ischemic attack	1.146 (0.620–2.120)	.663	0.977 (0.520–1.838)	.943	0.812 (0.616–1.070)	.140	0.779 (0.588–1.033)	.082	1.097 (0.802–1.502)	.562	1.061 (0.762–1.478)	.725
Intracerebral bleeding	1.416 (0.729–2.750)	.305	1.454 (0.740–2.857)	.278	1.679 (1.181–2.386)	**.004**	1.497 (1.048–2.137)	**.027**	1.736 (0.930–3.238)	.083	1.826 (0.965–3.456	.064
Gastrointestinal bleeding	1.325 (0.821–2.138)	.250	1.302 (0.803–2.112)	.285	1.158 (0.949–1.413)	.148	1.048 (0.856–1.285)	.648	1.647 (1.253–2.164)	**<.001**	1.387 (1.046–1.839)	**.023**
Transfusion of blood constituents	0.955 (0.880–1.036)	.265	0.923 (0.842–1.012)	.089	1.012 (0.967–1.060)	.600	0.876 (0.834–0.921)	**<.001**	0.932 (0.877–0.991)	**.025**	0.848 (0.794–0.906)	**<.001**
Pericardial effusion or hemopericardium	1.140 (1.016–1.278)	**.026**	1.105 (0.982–1.244)	.098	1.334 (1.260–1.411)	**<.001**	1.264 (1.193–1.340)	**<.001**	1.392 (1.298–1.492)	**<.001**	1.275 (1.185–1.371)	**<.001**
Hemopericardium	0.825 (0.609–1.118)	.214	0.793 (0.580–1.084)	.146	1.307 (1.119–1.527)	**.001**	1.149 (0.980–1.346)	.087	1.248 (1.041–1.495)	**.016**	1.113 (0.922–1.344)	.266
Pericardial effusion	1.193 (1.054–1.349)	**.005**	1.159 (1.021–1.314)	**.022**	1.330 (1.253–1.413)	**<.001**	1.275 (1.199–1.356)	**<.001**	**1**.407 (1.306–1.515)	**<.001**	1.295 (1.198–1.399)	**<.001**
Ventricular flutter/fibrillation	1.181 (0.994–1.403)	.059	1.171 (0.983–1.394)	.077	1.202 (1.091–1.324)	**<.001**	1.200 (1.087–1.324)	**<.001**	1.356 (1.150–1.600)	**<.001**	1.365 (1.150–1.620)	**<.001**
Angina pectoris	0.906 (0.748–1.097)	.313	0.935 (0.770–1.136)	.501	0.825 (0.772–0.883)	**<.001**	0.822 (0.767–0.880)	**<.001**	0.739 (0.687–0.795)	**<.001**	0.701 (0.648–0.759)	**<.001**
Ventricular tachycardia	1.007 (0.911–1.113)	.890	0.995 (0.899–1.102)	.929	1.164 (1.110–1.221)	**<.001**	1.122 (1.069–1.178)	**<.001**	1.084 (1.017–1.155)	**.014**	1.040 (0.972–1.112)	.253
Atrioventricular block	0.981 (0.856–1.125)	.784	0.985 (0.858–1.132)	.834	1.146 (1.068–1.230)	**<.001**	1.132 (1.053–1.217)	**.001**	1.199 (1.108–1.298)	**<.001**	1.096 (1.009–1.192)	**.031**
Treatment												
PCI	1.758 (1.336–2.314)	**<.001**	2.111 (1.575–2.828)	**<.001**	1.244 (1.132–1.368)	**<.001**	1.165 (1.055–1.287)	**.003**	1.190 (1.097–1.291)	**<.001**	1.011 (0.925–1.104)	.809
Coronary artery bypass graft	1.225 (0.369–4.070)	.740	1.193 (0.359–3.972)	.773	0.648 (0.450–0.934)	**.020**	0.555 (0.382–0.807)	**.002**	0.678 (0.452–1.018)	.061	0.587 (0.381–0.904)	**.016**
TAVR	‐	**‐**	‐	**‐**	‐	**‐**	‐	**‐**	‐	**‐**	‐	**‐**
Percutaneous edge‐to‐edge mitral regurgitation valve repairs with the MitraClip® implantation	‐	**‐**	‐	**‐**	‐	**‐**	‐	**‐**	‐	**‐**	‐	**‐**
Heart valve surgery	1.036 (0.638–1.680)	.887	1.076 (0.659–1.758)	.769	0.965 (0.788–1.181)	.730	0.894 (0.727–1.100)	.289	0.717 (0.548–0.939)	**.015**	0.635 (0.478–0.843)	**.002**
Implantation of pacemaker	0.617 (0.474–0.803)	**<.001**	0.618 (0.473–0.808)	**<.001**	0.818 (0.725–0.923)	**.001**	0.820 (0.725–0.929)	**.002**	0.931 (0.817–1.060)	.278	0.875 (0.761–1.006)	.061
Implantation of CRT	0.612 (0.184–2.034)	.423	0.599 (0.180–1.995)	.404	1.222 (0.772–1.935)	**.393**	1.201 (0.753–1.917)	.442	1.488 (0.976–2.267)	.064	1.077 (0.693–1.674)	.742
Implantation of implantable cardioverter‐defibrillator	0.704 (0.615–0.806)	**<.001**	0.703 (0.613–0.807)	**<.001**	0.834 (0.790–0.881)	**<.001**	0.798 (0.755–0.844)	**<.001**	0.887 (0.828–0.951)	**.001**	0.829 (0.771–0.892)	**<.001**
Catheter ablation for the treatment of cardiac arrhythmias	1.165 (0.976–1.390)	.090	1.170 (0.978–1.400)	.085	1.625 (1.445–1.828)	**<.001**	1.640 (1.455–1.850)	**<.001**	1.535 (1.322–1.781)	**<.001**	1.489 (1.275–1.738)	**<.001**
Ventricular assist device	0.938 (0.801–1.097)	.422	0.910 (0.773–1.071)	.256	1.265 (1.145–1.397)	**<.001**	1.101 (0.994–1.219)	.066	1.475 (1.227–1.774)	**<.001**	1.384 (1.144–1.673)	**.001**
Heart transplantation	0.865 (0.759–0.986)	**.030**	0.815 (0.710–0.937)	**.004**	0.867 (0.818–0.919)	**<.001**	0.705 (0.662–0.751)	**<.001**	0.754 (0.684–0.831)	**<.001**	0.642 (0.577–0.714)	**<.001**
Pericardial puncture	1.062 (0.842–1.340)	.613	1.037 (0.819–1.314)	.761	1.150 (1.017–1.301)	**.026**	1.144 (1.008–1.298)	**.037**	1.427 (1.241–1.640)	**<.001**	1.265 (1.092–1.464)	**.002**

*Note*: The first 5‐year cycle (2005–2009) was determined as the reference cycle. Bold values indicate statistical significance at *p* < .05 (two‐sided).

Abbreviations: CI, confidence interval; CRT, cardiac resynchronization therapy; EMB, endomyocardial biopsy; MACCE, major adverse cardiac and cerebrovascular events; OR, odds ratio; PCI, percutaneous coronary intervention; TAVR, transcatheter aortic valve replacement.

^a^ Adjusted for age, sex, obesity, cancer, heart failure, coronary artery disease, hyperlipidemia, chronic obstructive pulmonary disease, essential arterial hypertension, renal insufficiency (GFR < 60 mL/min/1.73 m²), diabetes mellitus, and atrial fibrillation/flutter.

## 4 DISCUSSION

The recommendations regarding the indications of EMB and the selection of patients for EMB were updated several times during the last decades. Nowadays, EMB is an established invasive procedure in daily cardiological routine.^24^, ^25^ Beside the most frequent use of EMB for the monitoring of heart transplant rejection, EMB plays a vital role with regard to establishing the diagnosis of several cardiac disorders, including HF of unknown reason, cardiomyopathies, myocarditis, drug‐induced cardiotoxicity, amyloidosis, other infiltrative and storage disorders as well as cardiac tumors.^24^, ^25^, ^26^, ^27^ Improvements in EMB equipment and techniques, in combination with significant progress regarding the histological and immune‐histochemical examinations, analysis, and validity of the EMB specimens and samples, resulted in a significant improvement in diagnostic precision and usefulness of EMB.^3^, ^25^ Previously published studies have shown that EMB is a widely safe procedure, and the changes overtime in interventional approach regarding localization of EMB (right vs. left ventricle) as well as procedure access (radial vs. femoral) seem not to have a significant impact on adverse events in patients undergoing EMB in large volume centers.^14^, ^28^ Nevertheless, data of large nationwide studies including low volume as well as mid‐ and high‐volume centers in respect to total annual EMB numbers are sparse; especially, for central Europe these data are missing. Thus, our study aimed to close this gap and to illustrate trends in EMB usage, rates of EMB‐related and nonrelated adverse in‐hospital events, and patients' accompanied treatment approaches.

The results of the present study can be summarized as follows:

(I) Overall, more than 67 000 EMB were performed in Germany between 2005 and 2019. Annual total numbers of EMB continuously increased during this investigated timeframe.
(II) Most patients who had to undergo EMB were most frequently aged between the 5th and 7th age‐decade of life.
(III) Patients who underwent EMB in the later 5‐year cycles were older and sicker, reflected by a higher dyspnoea level in co‐prevalence with an aggravated comorbidity profile.
(IV) Major complications decreased from 2005–2009 to 2015–2019, whereas prevalence of minor complications increased during the same period. While the decrease in major complications was regardless of age, the increase of minor complications was observed in patients aged 30–59 years but not in the younger and older age group.
(V) While MACCE rate increased overtime, driven by an increase of ischemic stroke, in‐hospital case fatality remained unchanged.
(VI) Total numbers of major complications and minor complications, as well as annual numbers of in‐hospital case‐fatality, increased with patients' age.
(VII) Since the annual numbers of EMB procedures, which were performed to establish the diagnosis of different cardiac disorders, increased overtime, the proportion of annual EMB procedures in patients with a history of heart transplantation as a primary cause of EMB decreased from 2005 to 2019.

In accordance with our study results, national trend analyses of the United States of America (US) observed a steady increase of the annual numbers of the in‐patient EMB procedures overtime.^29^ Similarly to the US, patients who underwent EMB were predominantly of male sex,^29^ whereas the median age of German patients with EMB was slightly higher than those of the US.^29^ In Germany, most patients who underwent EMB were frequently aged between the 5th and 7th age‐decade.

Our study results revealed important changes in the patient characteristics and comorbid profile of patients who underwent EMB overtime during the observational period (2005–2019), which might be partly the result of and explained by the expanded spectrum of indications evoked by adaptations in respect of the guideline recommendations to perform EMB in an increasing number of patients with unclear cardiac diseases.^24^, ^25^, ^26^, ^27^ In this context, patients who underwent EMB later during the 15‐year observational period were more often aged ≥70 years and showed an aggravated comorbid profile. In line with the suggestion that primarily the change in guideline recommendations^3^, ^24^, ^25^, ^26^, ^27^ might explain this shift, the proportion of EMB in patients with unclear acute cardiac disease manifestations increased. In contrast, the proportion of planned control EMB in patients with a history of heart transplantation decreased. Remarkably, it is well known that both complication and case‐fatality rates were substantially higher in EMB procedures in patients with native hearts (and acute and/or unknown cardiac diseases) compared to heart‐transplanted patients with control EMB.^29^, ^30^ In this context, we observed a substantial increase in the total numbers of major and minor complications and annual numbers of in‐hospital case‐fatality with patients' age. However, taking the age shift and aggravated comorbid profile of the patients undergoing EMB into account, major complications decreased from the timeframe 2005–2009 in comparison to the period 2015–2019. In contrast, the prevalence of minor complications increased during the same period. While the decrease in major complications was seen regardless of age and therefore, in all age groups, the increase regarding the occurrence of minor complications was observed in patients aged 30–59 years only.

The results of our study suggest an increased safety of EMB on a nationwide scale, as reported for smaller cohorts in the past.^12^, ^13^, ^14^, ^15^, ^25^ Despite an annually increasing vulnerability of the patient group (with older age and aggravated comorbidity profile) undergoing EMB in combination with a decreasing proportion of planned control EMB in patients with a history of heart transplantation, the technical progress with improvements mainly driven by the use of smaller and more flexible bioptomes result in lower complications rates and might play key roles for this improvement of safety outcomes.^13^, ^31^ Besides these technical improvements, the increasing annual numbers of EMB indicated for increased interventionalists' experience regarding EMB in later years of the observational period, which might also influence the safety outcomes beneficially.^3^, ^12^, ^24^, ^25^, ^32^, ^33^, ^34^, ^35^ This is in line with previously published studies, demonstrating that higher EMB‐procedure volumes were associated with lower complication rates after/peri‐procedural.^3^, ^12^, ^25^, ^35^ However, the annual number of EMB per operator, which is required to maintain the procedural skills, is still under debate. Recently, it has been suggested that the procedures per operator should exceed at least 20 procedures per year, but in other recommendations of the appropriate medical societies at least 50 procedures per operator per year.^3^, ^25^

Remarkably, despite MACCE rate increase in our study overtime primarily driven by the increase of ischemic stroke, the in‐hospital case‐fatality revealed unchanged after adjustment for age, sex, and comorbidities. While the increase in myocardial infarction and ischemic stroke overtime might be partly attributed to the higher age and aggravated comorbid profile of the patients undergoing EMB in later years, the increasing frequency of ischemic stroke might also be explained by higher numbers of left ventricular biopsies. Although it has been reported that both left ventricular EMB and right ventricular EMB are safe procedures with similar major complication rates if performed by experienced interventionalists,^13^, ^14^ ischemic stroke was reported as major complication in 0.3%–0.7% of studies in high‐volume centers when left ventricular EMB was performed.^13^, ^14^ In contrast, our real‐world data of all‐comers included in the German nationwide inpatient demonstrated a prevalence of ischemic strokes in patients who underwent EMB 2015–2019 of 1.5% (of all hospitalizations with performed EMB procedures in Germany). The high prevalence of ischemic stroke about EMB might be, on the one hand, driven by increasing numbers of left ventricular EMB, but on the other hand, driven by increasing patient age. In addition, the differences regarding the prevalence of the detected peri‐procedural ischemic strokes might be the result of higher complication rates in hospitals with low EMB numbers since the German inpatient sample included all hospitalizations of patients with EMB regardless of EMB center volume, which is an important difference regarding studies of single high‐volume centers.

Remarkably, we detected distinctly higher proportions regarding the combined outcomes of major and minor complications based on the present definitions of our study than in already published studies.^13^, ^14^ The higher rates of major and minor complications as combined outcomes in our study compared to other studies might be primarily attributed to differences in the definitions of the combined outcomes minor and major complications in the different studies. Additionally, due to the nature of the administrative data structure of the German nationwide inpatient sample, we could not verify that all of the peri‐procedural adverse in‐hospital outcomes and treatments were directly EMB related, but might also be the result of aggravation of the underlying disease, especially the performed interventional and surgical treatment, which were included in the combined outcomes of major and minor complications.^13^, ^14^, ^31^ Nevertheless, the primary objective of the present study focusing on the identification of time trends regarding outcomes and safety of the EMB interventions with decreasing major complications in recent years could be impressively established.

In this context, it must be mentioned that increasing numbers of EMB performed in patients with unclear cardiac diseases in combination with low left‐ventricular output lead to a higher number of implanted ventricular assist devices in the patients who underwent EMB during the hospitalization 2010–2019 compared to those treated between 2005 and 2009. In contrast, heart transplantation surgeries were less often performed during later years in patients who underwent EMB, which might be explained by the German organ transplant scandal with stagnating cardiac transplant numbers in later years.^36^, ^37^

Since EMB is in most hospitals only performed in a small number of patients with the exception of high‐volume centers,^3^, ^25^, ^38^ analyses of large cohorts including patients with EMB treated in high‐, mid‐, and low‐volume centers and especially of nationwide inpatient samples are of outstanding interest to identify important trends on performed procedures, patient characteristics and complications of patients who had undergo EMB.^16^, ^39^

## 5 LIMITATIONS

Certain limitations regarding our study require consideration: First, the study results are based on ICD and OPS discharge codes of inpatients. This might lead to incomplete data based on under‐reporting/under‐coding. Second, data about the administration of medications are not available in the data set of the Federal Statistical Office of Germany. Third, we could not provide follow‐up data. Fourth, we could not distinguish between left and right ventricular EMB as well as ultrasound‐guided versus fluoroscopy‐guided EMB. Fifth, the exact timing and course of adverse events/complications during hospitalization (i.e., whether it was present on admission or a complication during the hospital stay) could not be determined, whereby EMB is a selective procedure that will in the vast majority, only be performed in stable settings and not in acute emergencies during adverse events. Thus, adverse in‐hospital events are presumable in the vast majority peri‐procedural complications.

## 6 CONCLUSIONS

Annual total numbers of EMB increased significantly in Germany between 2005 and 2019. Although patients with EMB in later years were in median older with an aggravated and more severe comorbidity profile, major complications decreased during the observational period.

## AUTHOR CONTRIBUTIONS

Karsten Keller, Sebastian Göbel, Lukas Hobohm, and Philip Wenzel were involved in the conception and design of the study and analysis and interpretation of the data. Karsten Keller and Sebastian Göbel wrote the first draft of the manuscript. All authors contributed to drafting and revising the paper critically for intellectual content and gave final approval of the version to be published and agreed to be accountable for all aspects of the work.

## CONFLICTS OF INTEREST STATEMENT

T. M. is PI of the DZHK (German Center for Cardiovascular Research). L. H. reports receiving lecture honoraria from MSD, Johnson & Johnson, and Boston Scientific. T. G. has received grant support (CARIMA study) and speaker's honoraria from Novartis, speaker's honoraria from Boehringer Ingelheim, Daiichi‐Sankyo, MSD, Pfizer— Bristol‐Myers Squibb, and Astra Zeneca. P. W. reports receiving consultancy and lecture honoraria from Abbot Vascular, Astra Zeneca, Bayer, Boehringer Ingelheim, Daiichi‐Sankyo, and Novartis. The remaining authors declare no conflict of interest.

## Data Availability

The statistics of this study was carried out on our behalf by the Research Data Center (RDC) of the Federal Bureau of Statistics (Wiesbaden, Germany) analyzing the nationwide inpatient sample (NIS) of Germany (source: RDC of the Federal Statistical Office and the Statistical Offices of the federal states, DRG Statistics 2005–2019, own calculations). All codes used in this study are publicly available online. The data used in this study are aggregated study analysis results provided by the RDC; thus, we had access to summarised results provided by the RDC, but no access to individual patient‐level data, which will not be made publicly available.
